# The complete chloroplast genome of Chinese medicinal herb *Cynanchum chinense* R. Br. (Apocynaceae) and its phylogenetic position

**DOI:** 10.1080/23802359.2021.1959434

**Published:** 2022-04-01

**Authors:** Guodong Chen, Xue Zhang

**Affiliations:** a Key Laboratory of Fertility Preservation and Maintenance of Ministry of Education, Ningxia Medical University, Yinchuan, China; b Breeding Base for State Key Laboratory of Land Degradation and Ecological Restoration in Northwest China, Key Laboratory for Restoration and Reconstruction of Degraded Ecosystem in Northwest China of Ministry of Education, School of Ecological and Environmental, Ningxia University, Yinchuan, China

**Keywords:** *Cynanchum chinense*, chloroplast genome, Chinese medicinal herb, phylogenetic analysis

## Abstract

*Cynanchum chinense* R. Br. is an indigenous Chinese medicinal herb. In this study, the complete chloroplast genome of *C. chinense* was reported for the first time. The genome was 158,615 bp in length, with a large single-copy region of 89,958 bp, a small single-copy region of 19,415 bp, and 2 inverted repeat regions of 24,621 bp. The genome consisted of 132 genes, including 87 protein-coding genes, 37 tRNA genes, and 8 rRNA genes. The overall GC content is 37.82%. Phylogenetic analysis based on 21 complete genomes indicated that *C. chinense* is most closely related to *Cynanchum auriculatum* and *Cynanchum wilfordii*.

*Cynanchum chinense* R. Br. belongs to *Cynanchum* of Apocynaceae, is a perennial twining herb, and widely distributed in China. The whole plant is pubescent throughout and the winding length can be up to 4 m. The leaf blade is broadly triangular-cordate, basal veins up to 9, lateral veins up to 6 pairs, glaucous abaxially. Inflorescences normally forked at first flower, peduncle to 6 cm, rachis to 10 cm. Corolla white, rotate to reflexed, glabrous. Corona tube cupular, as long as gynostegium, margin with 5 threadlike lobes as long as corolla lobes alternating with 5 short rounded lobes, interior with 5 shorter, threadlike appendages. The juice contained in the aerial part of *C. chinense* is used for the treatment of warts (Liu et al. 2006), as well as the decoction of all parts can be used as Chinese medicinal herb to treat colds and chills (Yu et al. 2015). However, the potential therapeutic actions and clinical applicability of *C. chinense* are still not studied fully. The chloroplast genome can provide valuable genetic resources for further mining the medical potentials of *C. chinense*, therefore, we assembled and characterized the complete cp genome sequences of *C. chinense* by whole-genome sequencing (WGS).

Voucher specimen for *C. chinense* was collected from Zhanqu Village, Yinchuan city, Ningxia Hui Autonomous Region, China (E106°11′18.42″; N38°26′53.20″), and deposited at the herbarium of Ningxia University (Xue Zhang; zhxue@nxu.edu.cn; accession number: ZWNA-CHG-20200961). Fresh leaves from a single individual were used to extract the total genomic DNA according to a modified mCTAB protocol (Li et al. 2013). The DNA library was constructed using Illumina TruSeq^TM^ Nano DNA Sample Prep Kit and then paired-end sequenced using Illumina NovaSeq 6000 platform in Shanghai Biozeron Biothchnology Co. Ltd, China. A total of 4.8 Gb Next-Generation Sequencing data were generated. The cp genome of *C. chinense* was assembled with NOVOPlasty v2.7.2 (Dierckxsens et al. 2016), and then closed the sequence gaps using SOAPdenovo GapCloser v1.12 (Luo et al. 2015).

The cp genome of *C. chinense* was 158,615 bp in length, containing a large single-copy (LSC) region (89,958 bp), a small single-copy (SSC) region (19,415 bp), and two inverted repeat (IR) regions (24,621 bp). The overall GC content of *C. chinense* cp genome was 37.82%, and in the SSC, LSC, and IR regions were 32.29%, 36.01%, and 43.32%, respectively. The cp genome contained 132 complete genes, including 8 rRNA genes, 37 tRNA genes, and 87 protein-coding genes.

In order to evaluate the phylogenetic position of *C. chinense* in the Apocynaceae, the cp genome sequences of 21 species were aligned by MAFFT v7.475 (Katoh and Standley 2013), including 19 Apocynaceae species and 2 outgroups (*Gentiana scabra* and *Rubia cordifolia*). A phylogenetic tree (maximum likelihood) was constructed by MEGA v11.0.4 with 1000 bootstrap replications (Tamura et al. 2021). The phylogenetic tree revealed that *C. chinense* was most closely related with *C. auriculatum* and *C. wilfordii* (Figure 1). This complete cp genome provides valuable genomic resources that will be used for future studies on genetic phylogeny and pharmacology of *C. chinense* and other species of Apocynaceae.

**Figure 1.**
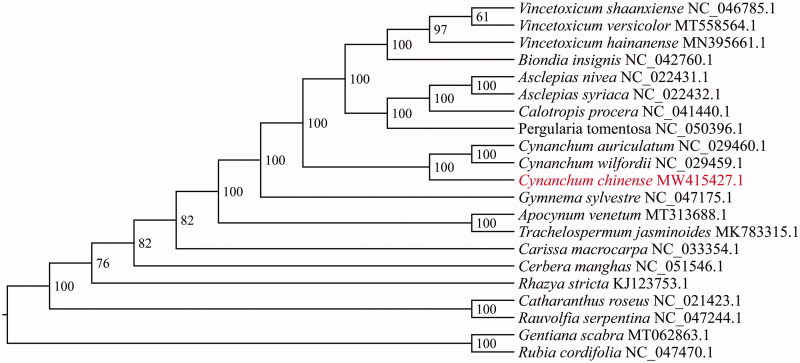
The maximum likelihood (ML) phylogenetic tree based on the complete chloroplast genome sequences of *C. chinense* and other related species. Bootstrap support values were shown next to the node.

## Data Availability

The genome sequence data that support the findings of this study are openly available in GenBank of NCBI at (https://www.ncbi.nlm.nih.gov/) under the accession number MW415427.1. The associated BioProject, SRA, and Bio-Sample numbers are PRJNA702020, SRR13721231, and SAMN17922146 respectively.
